# Histological Peripheral Margins and Recurrence of Melanoma In Situ Treated with Wide Local Excision

**DOI:** 10.1155/2020/8813050

**Published:** 2020-10-29

**Authors:** Francisco S. Moura, Lucy E. Homer, Stuart W. McKirdy

**Affiliations:** Department of Plastic & Reconstructive Surgery, Royal Preston Hospital, PR29HT, Fulwood, UK

## Abstract

**Background:**

The incidence of melanoma in situ (MIS) is increasing faster compared to invasive melanoma. Despite varying international practice, a minimum of 5 mm surgical excision margin is currently recommended in the UK. There is no clear guidance on the minimum histological peripheral clearance margins.

**Aim:**

This study compares the histological peripheral clearance margins of MIS using wide local excision (WLE) to the rate of recurrence and progression to invasive disease.

**Methods:**

A retrospective single-center review was performed over a 5-year period. Inclusion criteria consisted of MIS diagnosis, ≥16 years of age, and treatment with WLE with curative intent. Those patients with a recurrence of a previous MIS or with a reported focus of invasion/regression were also included. Clinicopathological data and follow-up were recorded.

**Results:**

167 MIS were identified in 155 patients, 80% of which were lentigo maligna subtype. Of patients with completely excised MIS on histology (>0 mm), 9% had recurrence with a median time to recurrence of 36 months. Three (1.8%) cases recurred as invasive disease. Age, MIS site, MIS subtype, and histological evidence of foci of invasion/regression did not predict recurrence nor progression to invasive disease (*p* > 0.05). The recurrence rate of MIS with a histological excision margin ≤3.0 mm was 13% compared to 3% in those with histology margins of >3.0 mm (*p*=0.049).

**Conclusion:**

A histological peripheral clearance of at least 3.0 mm is advocated to achieve lower recurrence rates. The follow-up duration should be reviewed due to the median recurrence occurring at 36 months in our cohort. Cumulative work on MIS needs to be collated and completed in a large multicenter study with a long follow-up period.

## 1. Introduction

Cutaneous melanoma is one of the fastest rising cancer diagnoses in recent years [1]. This is owed to an aging population and increased exposure to risk factors including sun exposure and immunosuppression [1]. Melanoma in situ (MIS) is a noninvasive lesion that accounts for up to 27% of all melanomas [2] and its incidence is increasing faster compared to invasive melanoma.

MIS is characterized by an increased number of atypical intraepidermal melanocytes [3,4]. This entity represents a precursor of invasive melanoma. Lentigo maligna (LM) is the most common subtype of MIS accounting for 79% to 83% of all MIS tumors [5, 6]. It is associated specifically with chronic exposure to ultraviolet radiation and primarily affects the head and neck region.

Surgical intervention by wide local excision (WLE) is the most widely used first-line therapy for MIS. However, no prospective RCTs have been performed, aiming to optimize margin control. Moreover, there is no international consensus on the optimal excision margin. The National Institute of Health and Care Excellence (NICE) guidelines in the UK currently recommends a minimum surgical excision margin of 5 mm [7] and recommend discharging the patient at the first outpatient clinic follow-up if the lesion has been histologically excised. The most recent guidance from the American Association of Dermatology (AAD) recommends a 5 to 10 mm surgical margin, recognizing that LM may require a larger than 5 mm margin [8]. Numerous reports have indicated that the current standards, particularly a surgical 5 mm margin, are inadequate for the management of MIS [9–22]. Importantly, there is no current guidance on the minimal histological margin clearance that should be achieved in the treatment of MIS when excised by WLE.

This study aims to evaluate the impact of the histological peripheral clearance margins of MIS on the recurrence and progression to invasive disease when treated with WLE. The novelty of this study is the heterogeneity of the study population. In addition to those patients that present with their first episode of MIS, prior studies have not incorporated cases that are treated as MIS in which the histology reports a potential regression and/or focus of invasion, as well as those that present with a recurrence of MIS.

## 2. Methodology

The principles of the Declaration of Helsinki were followed, and given the retrospective nature of this study, no formal patient consent was required. Royal Preston Hospital is the tertiary center for plastic surgery for the Lancashire region in the UK serving a population of 1.7 million people. All patients with a diagnosis of melanoma in this region of the UK are referred to our center. A single-center retrospective study was carried out to analyze patients with a diagnosis of MIS treated by wide local excision alone at our unit over a 5-year period between 1 January 2009 and 1 January 2014. Selection criteria included patients above the age of 16 years with a pathological diagnosis of MIS. These patients were identified through our pathological database using a SNOMED pathology code (M87422). Patients with primary MIS, recurrence of MIS, or a reported possible focus of invasion or regression (but treated primarily as MIS) were included in this study. In our unit, patients with MIS are treated with WLE only and not with Mohs micrographic surgery (MMS). Exclusion criteria included any patient that was not treated with curative intent or that had any other forms of treatment such as radiotherapy or other topical treatments. Further exclusion criteria included those which had a simultaneous diagnosis of invasive melanoma at the same site and were treated instead as an invasive disease.

Patient case notes (including operation notes, follow-up letters, and histopathological reports) were all carefully studied by two authors (FM and LH). Clinicopathological data collection comprised patient demographics, anatomical site of the lesion, melanoma subtype, evidence of invasion or regression in histology, histological excision margins, number of surgical interventions to achieve histological clear margins, recurrence, and progression to invasion.

Generally, WLE is carried out with a 5 mm surgical margin in our unit as per national guidelines [7]. Histological analyses of MIS specimens were performed as per the standard set by the Royal College of Pathology (RCPath) [23]. The specimens are fixed with formalin and bread sliced. The thickness is dependent on the size of the specimen. Typically, they are sliced into 3-4 mm thick cuts, and the nearest margins are further transversely sliced and examined. A constellation of morphological criteria is used to diagnose MIS including observation of a contiguous (lentiginous) growth of atypical melanocytes in the basal layer, with or without some pagetoid ascent. Immunohistochemistry using Melan-A antibody staining aids in the diagnosis of MIS.

Follow-up was recorded as the time of discharge from the clinic after the WLE. As per UK guidance [7], most patients (regardless of the first episode, recurrence, histological evidence of foci of invasion/regression, or histological clearance margin) with complete histological excision were discharged from the clinic at their first appointment at approximately 6 weeks to 12 weeks postoperatively. At discharge, patients are educated and strongly advised to contact our center if there are any concerns regarding recurrence. Patients are not discharged if they have a preference to continue follow-up in the clinic and have other lesions that require close monitoring or issues with delayed wound healing. A 5-year period following all patients' discharge was examined to check for possible recurrences. Recurrence was defined as those patients that had a further biopsy-proven pathological diagnosis of MIS at the same site.

To analyze the relationship between recurrence rate and the histological peripheral clearance, subjects were categorized into the following groups as proposed by the Cancer Outcomes and Services Dataset in the UK (advised by the RCPath, UK, and supported by the NICE, UK) [23]:

- Clearance by more than 5 mm
- Clearance at or by more than 1 mm, but less than or equal to 5 mm
- Clearance by less than 1 mm, but the tumor does not reach the margin
- Margin involved

To identify a minimum peripheral histological clearance margin associated with a lower recurrence rate, the peripheral margins of all subjects and their recurrence rates were analyzed. This commenced with a 1 mm peripheral margin and gradually increased by 1 mm until a statistically significant (*p* < 0.05) difference in recurrence was noted.

Statistical analyses were performed using Fisher's exact test for analyzing contingency tables and Kruskal–Wallis for comparison of nonparametric medians. These were carried out on StatsDirect statistical software (version 3.1.2), with a statistical significance at *p* < 0.05.

## 3. Results

During the 5-year study period, 155 patients with 167 cases of MIS met the inclusion criteria. The cohort had a mean age of 72 years (range 36–95 years) of which 72 (46%) patients were male. The median follow-up time was 8 months (interquartile range (IQR) 3–30 months).

**Figure 1:**
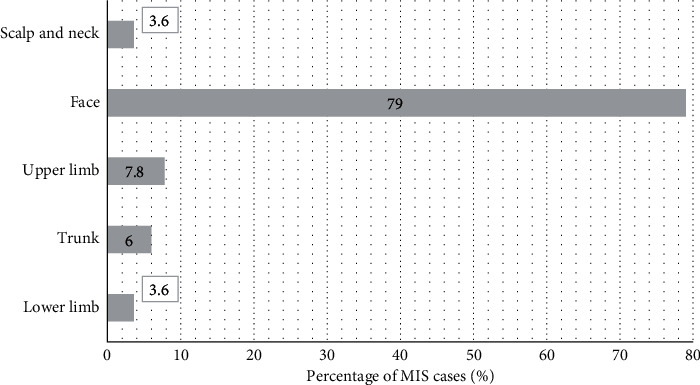
Percentage of MIS cases per body region. No statistically significant difference between gender and body site of MIS was identified (*p*=0.14).

**Figure 2:**
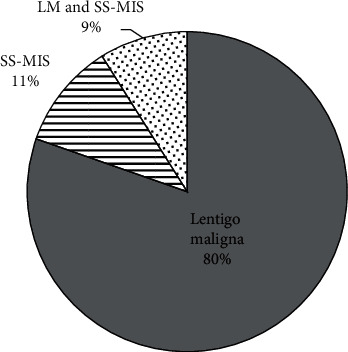
Distribution of different subtypes of MIS. LM: lentigo maligna; MIS: melanoma in situ; SS-MIS: superficial spreading melanoma in situ.

**Figure 3:**
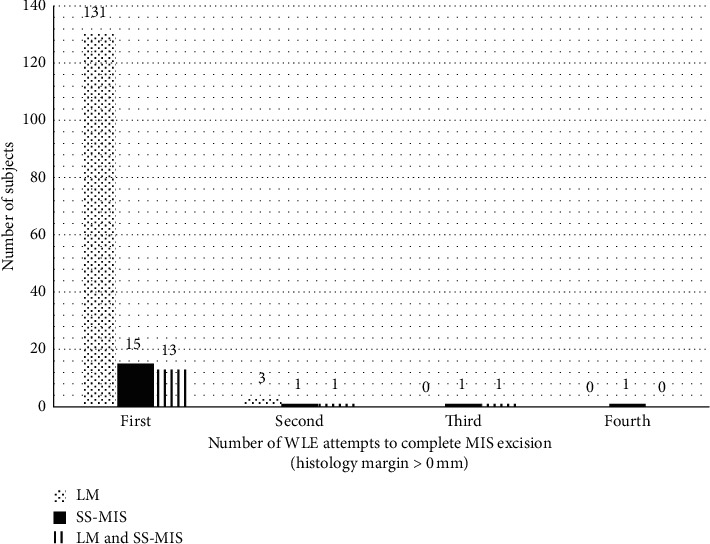
Number of WLE attempts to excise MIS (histology clearance >0 mm) per subtype. LM: lentigo maligna; MIS: melanoma in situ; SS-MIS: superficial spreading melanoma in situ.

The most common site of MIS was the head and neck (138 cases, 83%) followed by the upper limb (13 cases, 8%) (Figure 1). The anatomical distribution did not vary with gender (*p*=0.14). Primary MIS represented 84% (140 cases) of subjects whilst recurrent cases were 5% (9 cases) (Table 1). Most MIS subtypes within our cohort were LM (80%, 134 cases) whilst the remaining were superficial spreading MIS (SS-MIS) (11%, 18 cases) or had evidence of both SS-MIS and LM (9%, 15 subjects) (Figure 2). 160 (96%) subjects were completely excised at first surgery (Figure 3).

**Figure 4:**
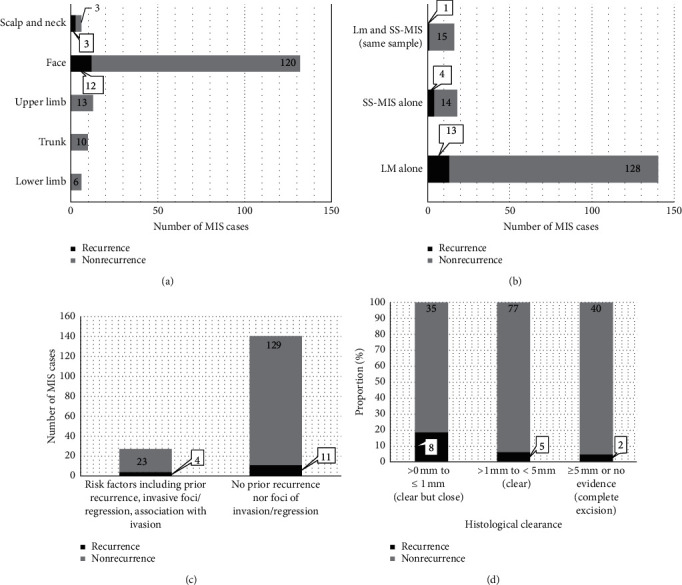
Comparison of recurrence and nonrecurrence following MIS excision. (a) Comparison of recurrence and nonrecurrence per body region. There was a statistically significant difference in the face compared to other sites of the body (*p*=0.004). (b) Comparison of recurrence and nonrecurrence per MIS subtype. There was no difference in recurrence depending on the MIS subtype (*p*=0.49). (c) Comparison of recurrence and nonrecurrence per presence (recurrent lesion, evidence of invasive foci, and regression) or absence of risk factors. There was no difference in recurrence with or without the presence of risk factors (*p*=0.27). (d) Comparison of recurrence and nonrecurrence per histological peripheral clearance margin. There was a reduced risk of recurrence with increasing histological peripheral margins (*p*=0.04). LM: lentigo maligna; MIS: melanoma in situ; SS-MIS: superficial spreading melanoma in situ.

The recurrence rate was 9% (15/167) with a median time to recurrence of 36 months (IQR 25–53) (Supplement Table 1). Three patients (1.8%) had recurrence with evidence of invasive melanoma. The only site of recurrence was the head and neck region with 15 cases (100%) (Figure 4(a)) (*p*=0.04). There was no difference in recurrence amongst LM (8%, 11/134) and non-LM (12%, 4/33) MIS subtypes (*p*=0.49) (Figure 4(b)). Similarly, there was no difference in the recurrence rate if a patient had a reported focus of invasion/regression and/or recurrence (*p*=0.27) (Figure 4(c)). The rate of recurrence decreased with increasing histological peripheral margins (*p*=0.037) (Figure 4(d)). The recurrence rate of lesions with a histological peripheral margin of ≤3.0 mm was 14% (13/103) compared to 3% (2/64) in those lesions with a histological margin of >3.0 mm (*p*=0.049). There was a statistically significant difference in median follow-up time between those with and without risk factors (28 months vs. 7.5 months, *p*=0.001). However, there was no statistically significant difference in follow-up between those with recurrence and recurrence-free (8 months vs. 13 months, *p*=0.5).

## 4. Discussion

The incidence of MIS is growing faster compared to invasive melanoma [24]. This is most likely due to improved public awareness, diagnosis, and increasing efficiency of referral services [24]. This is particularly relevant to countries such as the UK with a significant population with Fitzpatrick 1–3 skin type [25] which increases the risk of developing cutaneous melanoma [26]. Even though skin types were not individually assessed in our dataset, it is important to recognize that our study population likely falls within Fitzpatrick 1–3 skin type. Therefore, this limits the generalizability of the results of this study, making it most applicable to those populations with lighter skin tones.

Sun exposure is one of the principal risk factors for cutaneous melanoma [27, 28]. Subsequently, the head and neck region, besides being more visible to patients and their relatives, is the most common site of MIS as seen within our study. All recurrences in our study were limited to the head and neck region. Yet again, this is explained by the increased exposure of the head and neck region to the patient and their relatives. Moreover, if most cases of MIS are in the head and neck region in the first instance, then the probability of a recurrence in this area is logically higher as well. Although 5 mm is the standard excision margin for MIS in the UK, the operating surgeon may be more forgiving when excising an MIS from a less cosmetically sensitive area such as a limb as compared to the face, thus increasing the clearance margins and reducing recurrences.

One of the most prominent findings of our study was the high rate of recurrence of 9%, especially compared to other studies evaluating MIS recurrence with WLE treatment (Table 2) [27, 29, 31, 32]. There are several potential explanations for this high recurrence rate. In this study, we have included heterogeneous cases (recurrence or evidence of regression or foci of invasion), whilst in other studies, this has not been the case. Yet, no statistically significant difference was noted in recurrence amongst those cases with risk factors (recurrence, evidence of regression, and/or foci of invasion) and those cases that were seemingly risk-free. Of note, the median follow-up duration for those with risk factors was longer which could mean that a recurrence could have been picked up on consultation if it had occurred before discharge. The difference in follow-up duration could be explained by factors including patients being followed up for other diseases, delayed wound healing, or patient and clinician preference. In addition to the inherent bias of a retrospective study, patients were not actively followed up for a set period and instead encouraged to self-present if they were concerned about a recurrence. As a result, the recurrence rate in our study could be underestimated, and similarly, the 36-month average time for recurrence in our study may be an overestimation. This could have been prevented if a clinical specialist had assessed all subjects regularly.

Many studies suggest that a 5 mm surgical margin is inadequate [9–22]. The AAD currently recommends a 5 mm–1 cm surgical margin for MIS [8]. Garcia et al. [13] reported a 13.1 mm mean surgical margin in their serial disk staged excisions with zero recurrences over three months of follow-up. However, one should recognize that MIS typically affects the head and neck area and that such a wide margin of excision is not always pragmatic. Despite no recurrences in their study [13], our data indicate a median recurrence at 36 months which raises the questions if our study populations are comparable and if future studies should encompass a longer follow-up period.

The surgical margins used for WLE for each subject were not reported in this study. Hence, no inferences can be made to compare the surgical excision margins with both the histological margins and the recurrence rate, even if these cases are generally known to be excised with a 5 mm surgical margin [7]. One could argue that a histological peripheral margin of 3.0 mm is broadly similar to a 5 mm surgical excision margin once the specimen shrinks postexcision. Yet, no reliable scientific conclusions can be derived from such a variable relationship. Furthermore, the subclinical extension of MIS adds to the complexity of MIS excision as it is not visible to the operating surgeon unless the surgeon uses MMS or reflectance confocal microscopy (RCM).

Some groups have questioned the need for 1 cm surgical margins for all types of MIS as per the AAD guidelines [8] and instead support that only the LM subtype requires this wider margin [33, 34]. LM has been reported to have differences in behavior and outcomes compared to non-LM MIS due to a tendency towards subclinical peripheral extension and difficulty of histological diagnosis when located in sun-damaged skin. A retrospective review of 192 cases of MIS found that LM required wider margins for complete excision than did non-LM MIS [31]. LM has a reported local recurrence rate of 5% by two years [9] and carries up to a 4.7% lifetime risk of developing an invasive melanoma [35]. Still, Kunishige et al. [22] propose that subclinical extension of LM and MIS is similar and, as a result, propose the use of 9 mm surgical margins for all subtypes of MIS disease. This is in keeping with our finding that no difference in recurrence rate was identified amongst different MIS subtypes (Figure 4(b)).

Most lesions, regardless of their subtype and other histological features, were successfully excised as per histological report (>0 mm) at the first attempt of WLE (Figure 3). Importantly, our data demonstrate that an increased histological margin results in a lower rate of recurrence (Figure 4(d)). A statistically significant reduction in recurrence rate is seen when histological clearance exceeds 3.0 mm from 14% recurrence down to 3% recurrence (*p*=0.049). The latter recurrence rate is comparable to recurrences in other studies, even when our dataset includes subjects with presumed risk factors, such as recurrence cases and foci of invasion or regression, on top of the typical primary cases of MIS (Table 1).

A Cochrane review has revealed that there is a lack of high‐quality evidence for the treatment of MIS and LM [32]. Despite this, studies have demonstrated that all MIS subtypes have a high incidence of invasive foci [35–37]. Megahed et al. [37] reported that 29% of all MIS subtypes had invasive tumors. Because of this, some reports [38] advocate the treatment of MIS as an early-stage invasive melanoma. Our results demonstrate no significant difference in recurrence amongst those with and without evidence of foci of invasion.

In the UK, the mainstay of treatment for MIS is WLE. MMS is an additional surgical option for MIS which is not routinely offered to patients with a diagnosis of MIS in the UK. Compared to WLE, MMS offers the possibility of total margin evaluation and has been associated with decreased rates of recurrence [33]. Other recent studies contradict these findings by suggesting no differences in overall survival, cancer-specific survival, and recurrence rates amongst patients treated with MMS and WLE [29, 39]. There is a misconception that MMS is more costly than WLE, but recent preliminary work [34] would imply that MMS is more cost-effective than WLE, particularly with an increased incidence of skin cancers [1]. Such findings are important to the National Health System in the UK which is a fully publicly funded healthcare system.

An area that warrants attention is that recurrence may relate to both a wide subclinical extension of atypical melanocytes and the limitations associated with histological margin assessment of WLE samples. RCM has, and will, become a more common adjunct to the clinical exam, dermoscopy, and histopathology assessment [40]. In MMS, the entire margin is examined whereas in standard pathological assessment this is reported to range from 0.5% to 5% [41]. Should the decision be to continue managing patients with MIS in the UK using WLE, a national consensus should be reached to either advocate a minimal histological clearance margin, a specific follow-up plan depending on said histological margins, or promote a more detailed histological analysis of specimens. We cannot overlook the increased workload that would result from the latter for pathologists. Otherwise, surgeons should be rational in employing a 5–10 mm surgical margin as suggested by the AAD [8] instead of the 5 mm UK guidance [7].

## 5. Conclusion

More robust pathways for patients with a diagnosis of MIS are required. This body of work supports that the histological margins, particularly when using WLE as the means to surgically remove MIS, play an important role in the surgical management of patients with MIS. This study endorses that UK guidelines should aim for a consensus for a minimum histological clearance when MIS is treated by WLE. Our data indicate that a minimum histological margin of at least 3.0 mm should be advocated to achieve lower recurrence rates of MIS. Besides, the length of follow-up should be revised given the potential risk of recurrence and risk of invasion, particularly if the histological margins fall short of 3.0 mm. This is the first study to compare the impact of different risk factors such as recurrence, invasive foci, and regression on recurrence. These did not have a statistically significant impact on the rate of recurrence if complete histological excision was achieved. We, therefore, emphasize the need for further research into the histological peripheral margins of MIS excised by WLE in which cumulative work must be collated and completed in a large multicenter study with a prolonged follow-up monitoring period.

## Figures and Tables

**Table 1:** Different lesion types of MIS.

Lesion type	Subjects (*n*)	% of MIS subjects
Primary	140	84
	(120 LM; 12 SS-MIS; 8 both LM and SS-MIS)	

Recurrent	9	5
	(7 LM; 2 both LM and SS-MIS)	

MIS with invasive foci or regression	18	11
	(7 LM; 6 SS-MIS; 5 both LM and SS-MIS)	
Average BT depth of MIS with reported foci of invasion	0.47 mm	

**Table 2:** Comparison of recurrence rates amongst studies using wide local excision.

Study	No. of MIS subjects	Recurrence rate (%)
Joyce et al. [26]	410	2.2
Nosrati et al. [29]	385	5.7
Hou et al. [30]	269	5.9
Akhtar et al. [31]	192	2.9
Current study	167	9.0

## Data Availability

The data pertinent to the recurrences are available in Supplementary Table 1. Additional information from the study is available upon reasonable request.

## Abbreviations

AAD: American Association of Dermatology
IQR: Interquartile range
LM: Lentigo maligna
MIS: Melanoma in situ
MMS: Mohs micrographic surgery
NICE: National Institute of Health and Care Excellence
RCM: Reflectance confocal microscopy
RCPath: Royal College of Pathology
SS-MIS: Superficial spreading melanoma in situ
WLE: Wide local excision.

## Supplementary Materials

Supplementary Materials Supplementary Table 1 (attached file) contains the histopathological details of the patient with recurrences.
