# Supplementary material for: Histological Peripheral Margins and Recurrence of Melanoma In Situ Treated with Wide Local Excision
Source: J Skin Cancer. 2020 Oct 29;2020:8813050. doi: 10.1155/2020/8813050 (PMC7644340; doi:10.1155/2020/8813050)
Supplement: Supplementary Materials — Supplementary Table 1 (attached file) contains the histopathological details of the patient with recurrences. [file 8813050.f1.docx]

| **No.** | **Age** | **MIS subtype** | **Location** | **Evidence of invasive foci/ regression?** | **Recurrent lesion?** | **Histological excision margin of final clearance**  **/ mm** | **No. of WLE to achieve clearance** | **Time to recurrence**  **/months** | **Invasive melanoma at recurrence** |
| --- | --- | --- | --- | --- | --- | --- | --- | --- | --- |
| 1 | 56 | LM | Pre-auricular | No | No | 0.3 | 1 | 67 | No |
| 2 | 66 | LM | Cheek | No | Yes | <1 | 1 | 11 | No |
| 3 | 70 | LM | Cheek | No | No | <1 | 1 | 36 | No |
| 4 | 59 | LM | Cheek | No | No | 1 | 1 | 99 | No |
| 5 | 36 | LM | Cheek | No | No | 1 | 1 | 83 | Yes (BT 0.65mm) |
| 6 | 65 | LM | Neck | No | Yes | 3 | 1 | 41 | No |
| 7 | 80 | SS-MIS | Cheek | No | No | 2 | 1 | 52 | Yes |
| 8 | 37 | SS-MIS | Temple | Regression | No | Clear | 1 | 30 | No |
| 9 | 65 | LM | Eyelid | No | No | <1 | 1 | 44 | No |
| 10 | 65 | SS-MIS | Neck | No | No | 1 | 1 | 15 | No |
| 11 | 65 | LM | Cheek | Invasive foci (BT 0.4mm) | No | 3 | 1 | 30 | No |
| 12 | 37 | LM | Cheek | No | No | <1 | 1 | 35 | No |
| 13 | 73 | SS-MIS | Cheek | No | No | 2 | 1 | 9 | No |
| 14 | 72 | LM | Scalp | No | No | 5 | 1 | 19 | Yes (BT 0.23mm) |
| 15 | 72 | LM | Cheek | No | No | 3 | 1 | 54 | No |

Supplement Table 1: Clinicopathological details of MIS subjects with post-excisional recurrence. Abbreviation: BT – Breslow Thickness.
